# Authors’ Reply: Advancing Insights Into Postoperative Sleep Quality and Influencing Factors

**DOI:** 10.2196/70168

**Published:** 2025-02-03

**Authors:** Chen Shang, Ya Yang, Chengcheng He, Junqi Feng, Yan Li, Meimei Tian, Zhanqi Zhao, Yuan Gao, Zhe Li

**Affiliations:** 1 Department of Critical Care Medicine Renji Hospital School of Medicine, Shanghai Jiao Tong University Shanghai China; 2 Department of Infection Control Renji Hospital School of Medicine, Shanghai Jiao Tong University Shanghai China; 3 School of Biomedical Engineering Guangzhou Medical University Guangzhou China; 4 Department of Critical Care Medicine Peking Union Medical College Hospital Beijing China; 5 Institute of Technical Medicine Furtwangen University Villingen-Schwenningen Germany

**Keywords:** sleep quality, wearable sleep monitoring wristband, intensive care unit, minimally invasive surgery, traditional open surgery

Thank you for your thoughtful feedback on our article, “Quantitative Impact of Traditional Open Surgery and Minimally Invasive Surgery on Patients’ First-Night Sleep Status in the Intensive Care Unit: Prospective Cohort Study” [1]. We appreciate your insights and would like to address the key points you raised [2].

## Baseline Sleep Conditions

We fully agree that baseline sleep quality is crucial. In our study design, we excluded participants with chronic sleep disorders based on preoperative medical history and medication use. The suggestion to incorporate sleep scoring tools like the Pittsburgh Sleep Quality Index is valuable, as it could help investigate the impact of surgery on sleep status in different patient populations. Inspired by your comments, continuous monitoring using wearable devices during the preoperative and intensive care unit (ICU) periods could provide valuable insights into how sleep is dynamically affected by different clinical treatment protocols.

## Pain Management and Analgesic Strategies

We completely agree that pain is one of the greatest “enemies” of sleep quality. Working in a surgical ICU in a teaching hospital, we use standardized protocols along with the Richmond Agitation-Sedation Scale and Numeric Rating Scale (NRS) to manage pain and sedation. Our sedation and analgesia goals were maintained with Richmond Agitation-Sedation Scale scores of –1 to 0, aiming for an NRS below 3 [3,4]. Our unpublished data showed that both the minimally invasive surgery and traditional open surgery groups demonstrated comparable NRS scores (mean NRS: 1.86, SD 0.76, vs 1.85, SD 0.62; *P*=.96). This consistency allowed us to isolate the effects of surgical methods on sleep.

## Wearable Device Limitations

Rapid screening and diagnosis of sleep disorders in ICU settings remain challenging. Our study aimed to explore the use of wearable devices for patients who were awake in the ICU, helping to reduce bias introduced by sedative medications. We agree that ICU-specific environmental factors affect all electronic monitoring tools. Examining how these environmental factors impact device accuracy would be a meaningful research topic. The development of more sophisticated algorithms—whether embedded in device software or integrated with artificial intelligence–based predictive models using clinical data—represents a promising direction for the field of medical engineering integration.

## Psychological Factors and Study Design

Lastly, we appreciate your emphasis on the psychological factors influencing sleep, such as preoperative anxiety and depression. These factors may indirectly affect sleep quality, and the reduced trauma and faster recovery associated with minimally invasive surgery could positively influence psychological well-being. Exploring dynamic and quantitative methods to assess psychological states, alongside studying the effects of clinical treatments and mind-body interventions, presents a valuable opportunity to uncover their relationships with clinical outcomes. Combining these approaches with dynamic sleep state monitoring provides technical support to illuminate both direct and mediating relationships, paving the way for large-scale controlled trials to enhance patient care.

Thank you again for your valuable feedback. We look forward to further discussions and advancing this important field.

## Abbreviations

ICU: intensive care unit
NRS: Numeric Rating Scale
